# Characterisation of Reproduction-Associated Genes and Peptides in the Pest Land Snail, *Theba pisana*

**DOI:** 10.1371/journal.pone.0162355

**Published:** 2016-10-05

**Authors:** Michael J. Stewart, Tianfang Wang, Bradley I. Harding, U. Bose, Russell C. Wyeth, Kenneth B. Storey, Scott F. Cummins

**Affiliations:** 1 Genecology Research Centre, Faculty of Science, Health, Education and Engineering, University of the Sunshine Coast, Maroochydore DC, Queensland, 4558, Australia; 2 St. Francis Xavier University, Antigonish, Nova Scotia, 5000, Canada; 3 Institute of Biochemistry & Department of Biology, Carleton University, 1125 Colonel By Drive, Ottawa, ON, K1S 5B6, Canada; Nanjing University, CHINA

## Abstract

Increased understanding of the molecular components involved in reproduction may assist in understanding the evolutionary adaptations used by animals, including hermaphrodites, to produce offspring and retain a continuation of their lineage. In this study, we focus on the Mediterranean snail, *Theba pisana*, a hermaphroditic land snail that has become a highly invasive pest species within agricultural areas throughout the world. Our analysis of *T*. *pisana* CNS tissue has revealed gene transcripts encoding molluscan reproduction-associated proteins including APGWamide, gonadotropin-releasing hormone (GnRH) and an egg-laying hormone (ELH). ELH isoform 1 (ELH1) is known to be a potent reproductive peptide hormone involved in ovulation and egg-laying in some aquatic molluscs. Two other non-CNS ELH isoforms were also present in *T*. *pisana* (*Tpi-ELH2* and *Tpi-ELH3*) within the snail dart sac and mucous glands. Bioactivity of a synthetic ELH1 on sexually mature *T*. *pisana* was confirmed through bioassay, with snails showing ELH1-induced egg-laying behaviours, including soil burrowing and oviposition. In summary, this study presents a detailed molecular analysis of reproductive neuropeptide genes in a land snail and provides a foundation for understanding ELH function.

## Introduction

Snail reproduction is controlled by a tetra neural central nervous system (CNS) [1,2] that coordinates the timely release and action of potent neuropeptides. In molluscs, there are several neuropeptides that have attracted particular research interest due to their conserved role in reproductive processes, including Ala-Pro-Gly-Try-amide (APGWamide), egg-laying hormone (ELH), and gonadotropin-releasing hormone (GnRH).

APGWamide, first described in a sea snail by Kuroki *et al*. [3], is encoded by a precursor that possesses multiple lysine-arginine (KR) cleavage sites, giving rise to the N-terminal amidated bioactive APGW tetra peptide. In *Lymnaea stagnalis*, APGWamide is known to regulate male reproductive functions, such as controlling penis eversion and inhibition of spontaneous muscular contractions of smooth muscle in the *vas deferens* [4,5], as well as spermiation in the Donkey’s ear abalone (*Haliotis asinina*) [6].

The ELH peptide has been identified in both *Aplysia* and *Lymnaea* (called caudodorsal cell hormone, or CDCH, in *Lymnaea*), as well as two oyster species and a limpet [7,8,9,10,11,12], while analogues have also been described outside of molluscs such as in the fruit fly (*Drosophila*; ovulin) [13] and the leech (*Theromyzon tessulatum*) [14]. In *Aplysia*, ELH regulates functions of the female reproductive system, namely those involved with ovulation and egg laying [1,15]. *Aplysia* ELH that is released from the neural bag cells stimulates prolonged excitation of neurons in the abdominal ganglion and at the same time diffuses into the hemolymph to act as an endocrine neurohormone (Geraerts et al., 1988). When ELH reaches the animal’s ovotestis, it causes contraction of the smooth muscle follicles, thereby facilitating the expulsion of the egg string (Dudek and Tobe, 1978). In addition, ELH suppresses feeding behaviour by acting on the buccal and cerebral ganglia, and generates head waving by acting on the pedal and cerebral ganglia (Shyamala et al., 1986). Injection of the CDCH into *Lymnaea* stimulates ovulation and egg mass formation [16].

For GnRH, functional roles have been documented throughout most animal phyla (reviewed by Roch et al., [17]). In vertebrates, GnRH induces the release of gonadotropins whereas in the invertebrates research is still ongoing to define its precise role in reproduction, or other physiological processes. GnRH peptides have been identified in a number of aquatic molluscan species and while their function has not been well characterised, synthetic GnRH induces steroidogenesis and stimulation of spermatogonia proliferation in the octopus (*Octopus vulgaris*), Japanese scallop (*Patinopecten yessoensis*) and Pacific oyster (*Crassostrea gigas*) [17,18]. In general, a distinct feature of all GnRH precursor proteins is a well conserved GnRH peptide pQHWSX_4_PGamide that is cleaved from the variable N-terminal GnRH associated peptide [17].

The transition onto terrestrial environments has meant that land snails have diversified from other gastropods, and this has been accompanied by key changes to reproduction including aspects of both courtship and egg-laying to accommodate the lack of water (e.g. placement of eggs underground). One species that has evolved particularly effective adaptations for survival in dry habitats is the Mediterranean land snail *Theba pisana* (Müller, 1774). The ability of this hermaphroditic species to survive long periods with little or no water has also probably led to its success as an introduced pest in many areas where it can multiply at prolific rates causing widespread damage to agriculture [19,20]. Unfortunately, at present we have a limited understanding of the molecular components involved in hermaphrodite snail reproduction, particularly since, unlike most dioecious animals, their anatomy involves more complex pathways to regulate control over reproductive metabolism.

In this study, we performed *de novo* transcriptome sequencing of *T*. *pisana* CNS connective sheath to find the ELH gene. Through bioinformatic analysis we also identified the target reproduction-associated genes, ELH, APGWamide, and GnRH. We demonstrate that these genes share significant amino acid sequence homology with other molluscs and further show quantitative distribution of each within the CNS, dart sac and mucus glands. *In vivo* bioassays using a synthetic ELH peptide confirms that ELH has a crucial role in land snail reproduction, eliciting rapid changes by stimulation of egg laying.

## Methods

### Ethics Statement

All use of animals for this research was approved and carried out in accordance with the recommendations set by the Animal Ethics Committee, University of the Sunshine Coast.

### Animals

*T*. *pisana* were obtained from Warooka, South Australia (34.9900° S, 137.3990° E). The animals were identified as *T*. *pisana* by the criteria described in the integrated snail management in crops and pastures [19]. Once in the laboratory, snails were kept in terrarium-meshed pens at 19°C, 30 percent humidity and a 12:12h light:dark cycle. They were fed weekly with cucumber and carrot. To determine snail maturity, they were classified into three groups based on visual inspection of their reproductive systems upon dissection; juvenile (no reproductive system), immature (underdeveloped reproductive system including small mucus and albumin glands), and mature (fully developed reproductive system including presence of darts, large mucus and albumen glands).

### Protein Comparison and Annotation

A *T*. *pisana* RNA-seq library was assembled from SRP056280, as described in Adamson et al. (2015) [21]. Gene annotations corresponding to molluscan reproductive proteins were selected for comparative analysis using the CLC main workbench v6.9 (CLCbio) and BLASTp search. Matched nucleotide sequences were translated using ExPASy translate tool (http://web.expasy.org/translate/) and returned protein sequences were processed with SignalP (http://www.cbs.dtu.dk/services/SignalP/) [22] to determine secretion. NeuroPred (http://neuroproteomics.scs.illinois.edu/cgi-bin/neuropred.py) [23] was used to predict cleavage sites, posttranslational modifications, and the presence of putative bioactive peptides. Schematic diagrams of protein domain structures were prepared using the Domain Graph (DOG, version 2.0) software [24]. Protein secondary structure predictions were made using PredictProtein (http://www.predictprotein.org/), and protein 3D models were built using the Assisted Model Building with Energy Refinement (AMBER) 14 [25], in which the molecular dynamic simulations were sampled every picosecond for a total of 250 nanoseconds.

Protein sequences from *T*. *pisana* were aligned against the predicted protein sequences for APGWamide, ELH and GnRH peptide that were obtained from NCBI and Veenstra [11]. Using the MEGA 6.0 platform and in-built programs ClustalW and the Gonnet protein weight matrix [26], multiple sequence alignment schematics were generated and visualised through LaTeX’s TeXShade package [27]. Phylogenetic trees for GNRH were constructed using MEGA6.0 and the neighbour-joining method [28] with 1000 bootstrap replicates.

### Reverse Transcription-Polymerase Chain Reaction (RT-PCR) and Gene Cloning

Total RNA was isolated from nine different *T*. *pisana* tissues (CNS ganglia, bursa copulatrix, bursa tract, dart sac, foot muscle, hepatopancreas, mucus glands, ovotestis and penis) collected from both mature (*n* = 10) and immature animals (*n* = 15) using TRIzol (Invitrogen) extraction methods. Total RNA integrity was analysed using a 1.2% agarose gel with formaldehyde and ethidium bromide staining. Total RNA was used as a template for complementary DNA (cDNA) synthesis using a QuantiTect kit (QIAgen, Limburg, Netherlands) as per the supplier’s instructions. PCR was carried out on template cDNA using REDTaq (Sigma-Aldrich, MO, USA) as per supplier’s instructions and including gene specific primers (50 pmol each; S1 Table). Cycling parameters were 94°C for 1 min, 45°C for 2 min, and 72°C for 3 min for 30 cycles. PCR products were separated with a 2% agarose gel (0.6x Tris-Boric acid EDTA, TBE; 0.2% ethidium bromide) prior to visualisation (Syngene, Cambridge, England). Amplicons obtained were purified from 2% agarose gels with a QIAquick spin PCR purification kit (QIAgen) as per supplier’s instructions. Purified PCR products were then ligated into a pGEM-T easy plasmid (Promega, WI, USA) and recombinant clones were sequenced at the Australian Genome Research Facility (AGRF, Brisbane).

### Whole mount *in situ* hybridisation (WMISH)

Digoxigenin (DIG)-labelled riboprobes designed against the 3ˊ untranslated region of the *Tpi-ELH1* gene (identified in the transcriptome) and actin positive control were prepared for WMISH as previously described [29]. CNS, dart sac and mucous gland tissues were collected from mature snails and fixed in 4% paraformaldehyde in 0.01M phosphate buffered saline (Sigma; 138 mM NaCl, 2.7 mM KCl, pH 7.4) overnight, then stored in 70% ethanol at 4°C. WMISH was then performed using DIG-labeled riboprobes with modifications according to [29]. For documentation, specimens were dehydrated by stepwise ethanol changes, cleared in benzyl benzoate: benzyl alcohol (2:1 v/v) and mounted in 70% glycerol. Tissues were then examined with a Leica M205A stereoscope and images were captured with a Leica DFC550 digital camera.

### LC-MS/MS Identification of ELH

To confirm the production of ELH, protein was extracted from pooled CNS and pooled mucous glands from both mature and immature *T*. *pisana* (*n* = 30). Tissues were homogenized in 0.1% trifluro acetic acid (TFA) in H_2_O at a ratio of 1 ml per 200 mg of tissue. Homogenized tissue was then sonicated with three pulses (15 s each) and centrifuged for 10 min at 5,000 xg at 4°C. Hydrophilic biomolecules were isolated using a C18 Sep-Pack Vac cartridge (5 g; Waters, Rydalmere, NSW, Australia) following manufactures instructions. Samples were lyophilized and then resuspended in 1 mL 0.1% TFA for RP-HPLC using a gradient of 0%-60% acetonitrile over 60 min (Agilent Zorbax 300 SB-C18 column, 4.8 mm x 150 mm and particle size of 5μm). Sample eluates were collected in 5 min fractions and biomolecules detected at wavelengths of 210 nm and 280 nm.

Fractions were analysed by LC-MS/MS on a Shimadzu Prominance Nano HPLC (Japan) coupled to a Triple-ToF 5600 mass spectrometer (ABSCIEX, Canada) equipped with a nano electrospray ion source. Approximately 6 μl of each extract was injected onto a 50 mm x 300 μm C18 trap column (Agilent Technologies, Australia) at 30 μl/min. The sample was de-salted on the trap column for 5 minutes using 0.1% formic acid (aq) at 30 μl/min. The trap column was then placed in-line with the analytical nano HPLC column (150mm x 75μm 300SBC18, 3.5um; Agilent Technologies, Australia) for mass spectrometry analysis. Linear gradients of 1–40% solvent B over 35 min at 300 nl/minute flow rate, followed by a steeper gradient from 40% to 80% solvent B in 5 min were used for peptide elution. Solvent B was held at 80% for 5 min for washing the column and returned to 1% solvent B for equilibration prior to the next sample injection. Solvent A consisted of 0.1% formic acid (aq) and solvent B contained 90/10 acetonitrile/ 0.1% formic acid (aq). The ion spray voltage was set to 2400V, declustering potential (DP) 100V, curtain gas flow 25, nebuliser gas 1 (GS1) 12 and interface heater at 150°C.

The mass spectrometer acquired 500 ms full scan TOF-MS data followed by 20 by 50 ms full scan product ion data in an Information Dependant Acquisition, IDA, mode. Full scan TOF-MS data was acquired over the mass range 350–1800 and for product ion ms/ms 100–1800. Ions observed in the TOF-MS scan exceeding a threshold of 100 counts and a charge state of +2 to +5 were set to trigger the acquisition of product ion, ms/ms spectra of the resultant 20 most intense ions. The data was acquired and processed using Analyst TF 1.5.1 software (ABSCIEX, Canada). Proteins were identified by database searching using PEAKS v6.0 (BSI, Canada) against a database of protein sequences predicted from the *T*. *pisana* connective sheath transcriptome library and protein database composed of known neuropeptides from molluscs [12].

### ELH Bioassay

Mature snails (*n* = 78, 6 per treatment group; 1.85g ± SD 0.25 g body weight) were selected at random and administered either 5 μl or 10 μl of synthetic ELH1 (China peptides; delivered via a 50 μl Hamilton syringe) at one of five concentrations: 10^−12^ M, 10^−9^ M, 10^−6^ M, 1.15x10^-4^ M or 10^−3^ M. All solutions were prepared in molluscan Ringer (30), and concentrations were selected based on previous studies in *A*. *californica*, that indicated that ELH1 is effective within this range to stimulate egg-laying [10]. Following injection, all snails were placed into individual 500 ml specimen jars 1/4 filled with damp loam. They were then held at ambient 18–21°C with a 12:12 h light/dark cycle. Changes in behaviour including burrowing and egg-laying were monitored every 10 min for up to 1 h post-injection and then every hour for 6 h, after which they were monitored every 24 h for up to 6 days post-injection. Negative controls included injection of 10 μl BSA (10μg/ml in Ringer) or molluscan Ringer solution [30]. Snails pierced with a syringe were also assessed without injection of any solution.

## Results

### Reproduction-Associated Gene Identification and Analysis

**Fig 1:**
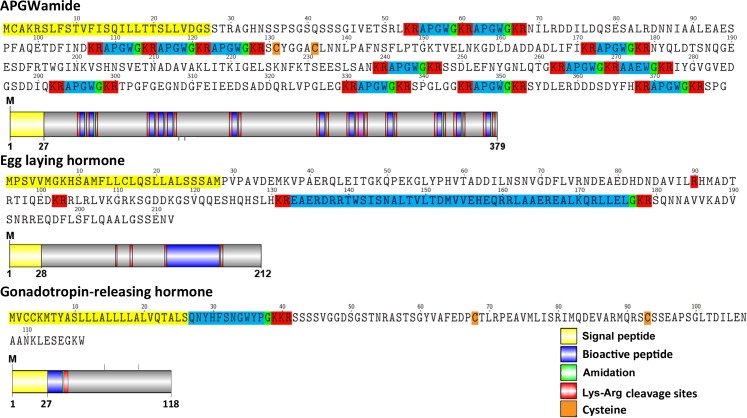
Molecular characterisation of *T*. *pisana* APGWamide, ELH and GnRH precursor proteins. An amino acid sequence and schematic representation is shown for each.

A transcript for APGWamide (*Tpi*-*APGW1*) was identified that encodes a precursor containing a 27-residue signal sequence and multiple regions for cleavage and release of a suite of APGWamide peptides (**Fig 1**). The Tpi-APGW precursor consists of 379 amino acids although no terminal stop codon was present and so is presumed to be partial-length. One transcript for ELH (*Tpi*-ELH) was identified that encodes for a single full-length protein precursor. The 212 amino acid *Tpi*-ELH precursor contains a 28-residue signal sequence and cleavage sites for the release of a single amidated ELH peptide containing 44 amino acids (**Fig 1**). A single transcript for a *T*. *pisana* GnRH precursor (*Tpi-GnRH*) was identified that encodes for a 116 amino acid precursor. The precursor contains a 26-residue signal sequence and cleavage sites for the release of an 11-residue GnRH peptide and 76-residue GnRH-associated peptide (**Fig 1**). The Tpi-GnRH precursor shows high amino acid homology in the GnRH peptide region with other molluscan sequences, especially to *A*. *californica* through conservation of Tyr at the N-terminus of the GnRH peptide (**S1 Fig**). Tpi-GnRH also shows typical GnRH-like characteristics suggesting an N-terminal pyroglutated Gln, and the presence of Ser, Gly and Trp which are found in the majority of GnRH family peptides [17].

**Fig 2:**
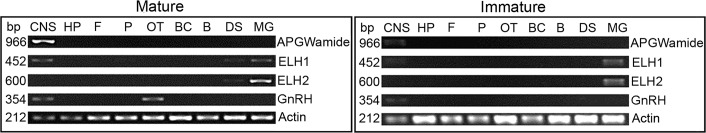
Representative RT-PCR gel showing tissue-specific expression of APGWamide, ELH and GnRH precursor genes in *T*. *pisana*. Tissues were derived from mature (*n* = 10) and immature (*n* = 15) snails. Tissues used include the: bursa tract (B), CNS (whole central nervous system ganglia), dart sac (DS), foot muscle (F), hepatopancreas (HP), mucous glands (MG), ovotestis (OT), penis (P). *Tpi-actin* was used as a positive control.

Tissue-specific expression of *APGW*, ELH and *GnRH precursor* genes was undertaken using RT-PCR with gene-specific primers on RNA derived from tissues of mature and immature *T*. *pisana* (**Fig 2**and **S2 Fig**). *Tpi*-*APGW* gene transcripts were present only within CNS tissue, of both mature and immature snails. The *Tpi*-*GnRH* gene was expressed in the CNS and ovotestis tissue of mature snails, and only the CNS of immature snails. A *Tpi*-*ELH* amplicon of the expected size was identified in the CNS and mucous gland of mature and immature snails (ELH1), and in the dart sac of mature snails. In addition, two larger amplicons were also identified, one from the dart sac of mature snails (ELH2), and another from the mucous gland of both immature and mature snails (ELH2).

### Analysis of ELH Genes

**Fig 3:**
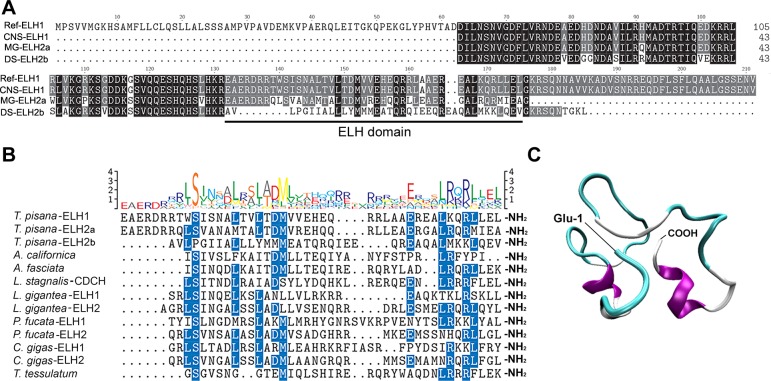
Molecular identification of *Theba pisana* egg laying hormone (ELH). (**A**) Comparative amino acid sequence alignment of the ELH region showing sheath ELH (Ref-ELH1) with ELH amplicons derived from the CNS, DS (ELH2) and MG (ELH3). Black shading represents identical residues, grey shading represents similar residues. The ELH peptide domain is shown. (**B**) Amino acid sequence and comparative amino acid sequence alignment of known ELH peptides. Sequence logo provides an overall view of sequence conservation with the scale bar indicating the degree of amino acid conservation. (**C**) Predicted structure of *T*. *pisana* ELH1. Purple represents alpha-helix, Thr8-Ile11, Lys38-Leu42; cyan turns, Gln1-Arg4, Ser12-Thr16, Thr19-Glu26, and Leu30-Arg34 white random coil.

All ELH amplicons were sequenced, showing that the CNS ELH1 amplicon corresponds to *Tpi*-*ELH* identified within the CNS transcriptome (Ref-ELH), whereas the larger amplicons retrieved from mucous gland and dart sac amplicons, turned out to be show distinct variations (ELH2 and ELH3) within the ELH peptide (**Fig 3A**). The *Tpi*-ELH precursor showed strong homology to other mollusc ELH precursors within the ELH peptide, primarily near the N- and C-termini. This conservation was also apparent when Tpi-ELH was compared with other identified ELH (**Fig 3B**). A predicted 3D structure of the ELH peptide shows that it likely consists of two short alpha helices (**Fig 3C**). The potential energy as a function of time during this molecular dynamics simulation (MDS) and the root mean-square deviation of atomic positions (RMSD) relative to this structure during the course of the MDS (**S3 Fig**), suggests it reached a family of stable conformations (RMSD<2Å). The representative structure of Tpi-ELH occurred at 106.60 ns into the MDS.

**Fig 4:**
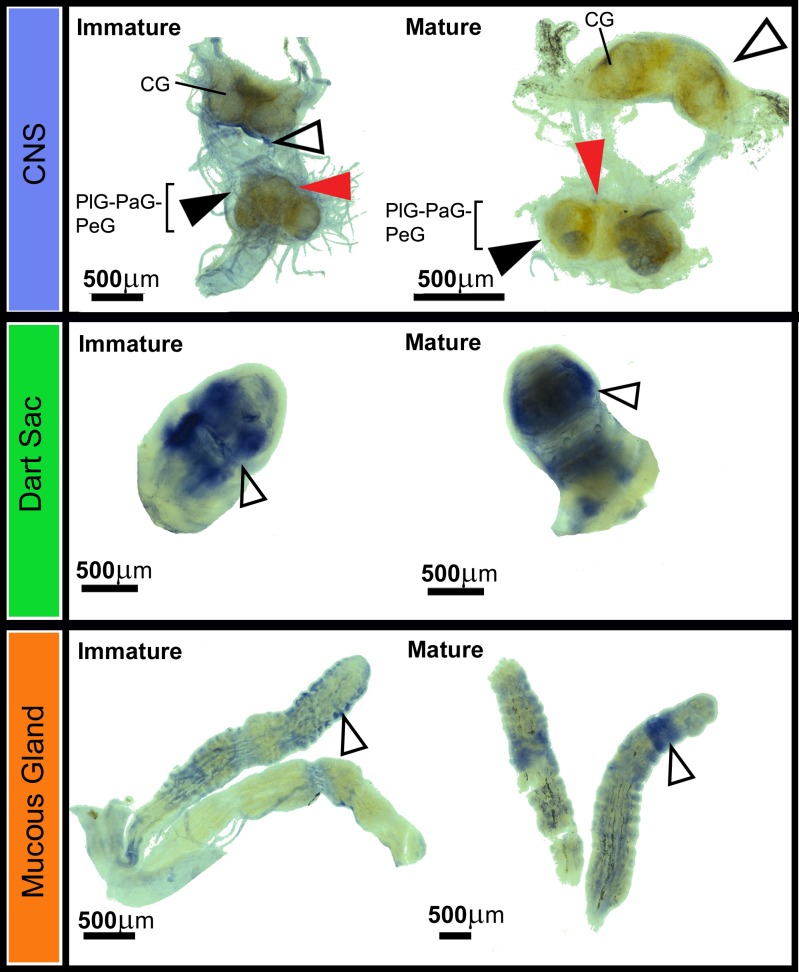
Whole-mount *in situ* hybridisation localisation of *Theba pisana ELH1* within mature and immature snail CNS, dart sac and mucous gland. Antisense DIG-labeled riboprobes show the location of *Tpi-ELH1* transcript. Open arrowheads, represent example expression in the dart sac and mucous gland, as well as the cerebral ganglia sheath. Black arrows highlight expression of *Tpi-ELH1* in the parietal/pleural/visceral ganglia region; the red arrowheads show location of the pedal ganglia.

Whole mount *in situ* hybridisation with an antisense *ELH1* DIG-labeled riboprobe was used to determine the spatial expression of *Tpi-ELH1* in mature *T*. *pisana* CNS, dart sac and mucous glands (**Fig 4**). In the CNS, *Tpi*-*ELH1* expression was observed in regions of the connective sheath, closest to the cerebral ganglia and procerebrum. *Tpi*-*ELH1* expression was also observed throughout the dart sac, whereas the mucous glands exhibited punctate expression throughout the acini, which are known to contain clusters of mucus-secreting cells. Negative controls displayed marginal background staining restricted to the outer proximity of the connective tissue. No background staining was observed in the mucous glands or dart sac.

**Fig 5:**
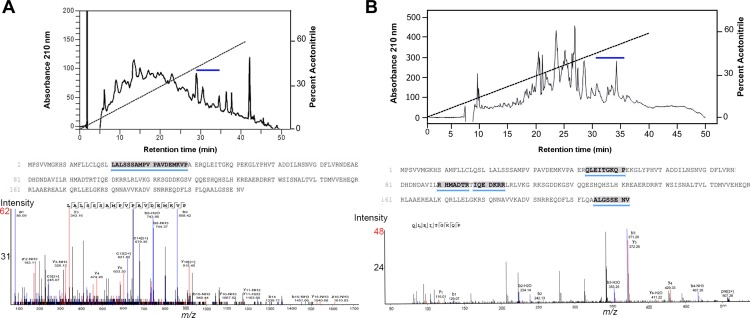
Proteomic evidence of ELH precursor peptides within protein extracts of *T*. *pisana* tissues. Representative RP-HPLC elution profiles, precursor sequence with matching peptide shown and MS/MS spectra for (**A**) CNS and (**B**) mucous gland. Blue line in chromatograms indicates where Tpi-ELH peptides eluted.

RP-HPLC was performed on CNS and mucous gland extracts to identify whether any of the *Tpi*-ELH precursors could be identified. Representative RP-HPLC elution profiles for CNS and mucous gland are shown in **Fig 5A and 5B**, respectively, where fractions between 20–40 min elution were further analysed by LC-MS/MS. A single peptide segment matching to the Tpi-ELH precursor was found in the CNS extract at 30–35 min, corresponding to the L_21_ALSSSAMPVPDEMKVP_39_ ([M+1H]^1+^ = 1610.83) (**Fig 5A**). Three different Tpi-ELH1 precursor peptide segments were identified from the mucous gland extracts, which also eluted within 30–35 min (**Fig 5B**), an example of which corresponds to Q_43_LEITGKQP_51_, ([M+2H]^2+^ = 507.28).

### Bioassays

Mature *T*. *pisana* were also analysed for egg-laying following injection of snails with either synthetic Tpi-ELH1 or control solutions **(Table 1)**. In Assay 1, within 60 min post-injection 100% of snails (*n* = 78) had recovered, as demonstrated by normal activity. No behaviour change, including egg-laying, was observed in negative controls (snails injected with Ringer solution, BSA, or injection with no solution) 24 h post-injection. By contrast, soil-burrowing behaviour was observed in 40.7% of snails treated with Tpi-ELH1 (*n* = 22), including a single Tpi-ELH1-treated snail (1.15x10^-4^M Tpi-ELH) that had produced an egg mass containing fertilised and non-fertilised eggs. In subsequent observations at 48 h and 72 h post-injection, an additional 5 snails from treatment group ELH 1.15x10^-4^ M had laid egg cordons. No mortality was observed in any of the treatment groups.

**Table 1:** Egg-laying bioassay following injection of synthetic Tpi-ELH1 and or negative controls.

Stimulus	*n*	Negative	Burrowing without Egg-laying	Burrowing with Egg-laying	Egg-laying only
**Assay 1**					
Injection (no solution)	6	6	0	0	0
Ringer (10μl)	6	6	0	0	0
BSA (10μg/ml, 10μl)	6	6	0	0	0
ELH (10-^3^M, 5μl)	6	6	0	0	0
ELH (10-^3^M, 10μl)	6	6	0	0	0
ELH (1.15x^-4^M, 10μl)	6	0	0	6	0
ELH (10-^6^M, 5μl)	6	1	5	0	0
ELH (10-^6^M, 10μl)	6	4	2	0	0
ELH (10-^9^M, 5μl)	6	0	6	0	0
ELH (10-^9^M, 10μl)	6	2	4	0	0
ELH (10-^12^M, 5μl)	6	4	2	0	0
ELH (10-^12^M, 10μl)	6	3	3	0	0
**Assay 2**					
Injection	6	6	0	0	0
ELH (1.15x^-4^M, 10μL)	30	11	0	16	3

Given that snails given 1.15x10^-4^M Tpi-ELH showed egg-laying, in Assay 2 snails (*n* = 30) were injected with only 1.15x10^-4^ M ELH **(Table 1)**. Again, no immediate egg-laying was observed, but by 24 h post-injection, 3 snails had exhibited egg-laying behaviour (burrowed into the soil), and 2 snails had laid eggs. After 48 h post-injection, another 13 snails had laid eggs, while 2 more egg masses were present at 72 h post-injection. Some egg masses were found above the top soil (*n* = 3). By day 6, 2 more snails produced egg masses before the experiment was terminated. Negative control (injection without solution, *n* = 6) produced no eggs or and snails did not exhibit burrowing or egg-laying behaviour.

## Discussion

In this study, we analysed the CNS of *T*. *pisana* to assess the presence of molluscan-like reproductive neuropeptide genes. Our *T*. *pisana* CNS RNA-seq data revealed the presence of two partial-length Tpi-APGW1 precursor isoforms (*Tpi-APGW1* and *Tpi-APGW2*). At 379 amino acids, and encoding twelve APGWamide peptides, Tpi-APGW1 was larger (by at least 72%) than the *Haliotis asinina* APGWamide precursor [31], and is approximately 29% larger than Tpi-APGW2. This variability was due to the inclusion of a single 111 amino acid insert in Tpi-APGW1 compared with Tpi-APGW2, and comparative to other molluscs, this makes it the largest known molluscan APGWamide precursor identified to date. Various analogues such as the related GWamide peptides (TPGWa, KPGWa and RPGWa) have been identified in the cuttlefish *Sepia officinalis* [32,33], the blue mussel *Mytilus edulis* [34], and the Pacific (*C*. *gigas*) and Pearl (*Pinctata fucata*) oysters [12], but these were not present in *T*. *pisana*. However, we did identify a single amidated peptide, AAEW, which is encoded in both Tpi-APGW isoforms. The *Tpi-APGW* gene was exclusively expressed within the CNS, a location that is consistent with other gastropods. Specifically, APGW has been reported to localise to the antero-medial region of the cerebral ganglia, and in part of the pedal ganglia [35,36,37,38]. Further work will be required to identify the full-length sequence of *Tpi-APGW* and characterise the role and function of both AAEWamide and APGWamide in *Theba*.

In molluscs, genes encoding the GnRH have previously been identified in the CNS tissues of *O*. *vulgaris* [39] and *Aplysia* [40]. Also, it was recently annotated from the draft genomes of the oysters *C*. *gigas*, and *P*. *fucata* [12] and the limpet *L*. *gigantea* [11]. Furthermore, numerous studies have also documented the presence (through immunolocalisation) and bioactivity of GnRH in a variety of other molluscan species [41,42,43,44,45,46,47], thus confirming the evolutionary conservation of GnRH amongst this diverse phylum. In this study, we identified a transcript encoding a full-length precursor for Tpi-GnRH within the sheath transcriptome with subsequent spatial expression analysis showing that it was also present in the ovotestis of mature animals. This location is not uncommon in molluscs as demonstrated in *S*. *officinalis* and *H*. *asinina* where the GnRH gene is also expressed in ovary [48,49]. Our study supports a role for GnRH in reproduction, and further supports the idea that regardless of the evolutionary distance among animal phyla, GnRH is an ancient peptide that consistently shows a role in the broad context of animal reproduction [17,48]. This contrasts with the diversity of GnRH-like isoforms, including AKH, corazonin and RPCH that have been found among the ecdysozoa, that have not yet been directly associated with reproductive processes [50,51].

The ELH gene is quite variable, making the bioactive peptide difficult to positively identify through traditional molecular tools. More recently, due to the relative ease with which nucleic acids can be sequenced, high-throughput transcriptome mining approaches have enabled researchers to identify putative neuropeptides, even within non-model animal species [12,52]. Thus, transcriptome mining is now a valuable tool for gene discovery and has been used effectively in a number of species, which is helpful for when a genome is unavailable [53]. For example, the oyster ELH genes were curated from *in silico* screening of the genomes of the Pacific oyster (*Crassostrea gigas*) and Pearl oyster (*Pinctata fucata*) [12].

*T*. *pisana ELH1* encodes a protein that shares the characteristic ELH preprohormone features, including a hydrophobic signal sequence, followed by a number of cleavage sites that can release a bioactive ELH peptide [54]. However, Tpi-ELH1 contains no homologous peptides within its precursor that have similarity to the α or β peptides of ELH as described within the *Aplysia* ELH precursor. These additional bioactive precursor peptides are recognised for their role in auto-excitation of *Aplsyia* ELH-containing neurons [55]. However, since the Tpi-ELH1 precursor contains predicted cleavage sites that could liberate at least 4 additional peptides, we presume that these could play similar roles to those described in *Aplysia*. The C-terminal peptide (SQNNAVVKADVSNRREQDFLSFLQAALGSSENV) is overall acidic, a feature that that is consistent with the C-terminal peptide of the *Aplysia* ELH precursor, known as the acidic peptide [56]. Comparing the primary structure of Tpi-ELH1 with other molluscan ELHs reveals conserved regions within the N- and C-termini, including amidation, thought to play an important role in receptor binding [9].

Accumulated genomic data have shown that the ELH gene is a member of a multigene family consisting of a small number of highly homologous genes that are expressed in a tissue-specific fashion [12,57]. There are also reports of multiple ELH-like peptides in the same precursor. For example, in the oysters *P*, *fucata* and *C*, *gigas*, the *ELH* gene precursor sequence encodes precursors that include two ELH-like peptides [58]. We have found that although *T*. *pisana* does not contain a precursor with multiple ELHs, it does have an additional two ELH genes, the *Tpi-ELH2* and *Tpi-ELH3*, which are expressed in non-neural tissues, as determined by tissue-specific RT-PCR. This has interesting parallels with the *Aplysia* atrial gland ELH-like peptide, the last major exocrine organ to make contact with eggs before they are laid [59]. It has been proposed that, under normal physiological circumstances, the atrial gland cannot secrete ELH into the hemocoel, and therefore does not play a direct role in egg deposition [60]. Instead, when the atrial gland peptides are injected into mature animals they can elicit egg deposition due to the direct action of the ELH-related peptides [61,62]. This suggests that the location of the atrial gland and its role may be more consistent with inducing stimulatory mating behaviour [63,64].

In our study, we found *Tpi-ELH1-3* in the dart sac and mucous gland of mature *T*. *pisana* (but only mucous gland of immature snails), thus implicating this hormone as a contributor to the proposed transfer of allohormones during ‘love’ dart shooting in helicid snails. The love dart acts as a syringe-like needle that facilitates the co-transference of a precoated mucous gland substance(s) at mating that increases paternity success [65,66,67,68,69,70,71]. No studies to date have clearly defined the exact biochemical makeup of the helicid love dart allohormone, although at least one component of the allohormone is known to stimulate contraction of the recipient bursa duct diverticulum, thereby ensuring that an increased proportion of its donor sperm are stored [72]. Therefore, the effect of Tpi-ELH1 and Tpi-ELH2 on bursa duct diverticulum contraction should be explored. However, the present study has established that synthetic Tpi-ELH1 could induce observable changes in egg laying behaviour *in vivo*. In *Aplysia*, ELH can stimulate egg laying in approximately 45 min post-injection [7]. The time taken for Tpi-ELH1-induced egg laying in *T*. *pisana* was much longer (180 mins to 24 h post-injection), and those animals that did exhibit egg laying produced immature eggs. This result may be due to isolation of individual snails during bioassays, or periods in which snails were in dormancy or in periods of inactivity resulting in slower snail recovery and mobilisation.

## Conclusions

Reproductive neuropeptides encompass a diverse class of cell signalling molecules that are produced and released via an endocrine regulated secretory route. In this study, we analysed a CNS transcriptome from the land snail *T*. *pisana*, and identified gene transcripts that encoded for three ELH peptides, the ELH1-3. Based on functional analysis, we have implicated a role for ELH1 in reproductive processes of *T*. *pisana*. Of significance, this is the first time that an ELH has been characterised at both the gene and functional level in a land snail. We also report other gene transcripts that have been implicated in reproductive processes in molluscs, including *APGW* and *GnRH*. It is conceivable that this information could now be used to develop a basis for understanding of reproduction in other terrestrial molluscs and develop ways for managing pest land snail populations through modulation of these genes.

## Supporting Information

S1 Fig Comparison and phylogenetic analysis of GnRH. (TIF)

S2 Fig Complete RT-PCR gel showing tissue-specific expression of APGWamide, ELH and GnRH precursor genes in *T*. *pisana*. Representation shown in **Fig 2**. Tissues used include the: bursa tract (B), CNS (whole central nervous system ganglia), dart sac (DS), foot muscle (F), hepatopancreas (HP), mucous glands (MG), ovotestis (OT), penis (P). *Tpi-actin* was used as a positive control.
(TIF)

S3 Fig ELH1 model. (A) Potential energy of ELH1 as a function of time during MD. The solid line is a running average over 50 ps. (B) Backbone RMSD during the same MD, compared to the lowest-energy conformation (the representative structure). ELH1 sequence: [p-] EAERDRRTWSISNALTVLTDMVVEHEQRRLAAEREALKQRLLELamide.
(TIF)

S1 Table Primers used for RT-PCR. (DOCX)
