# Supplementary figures and images for: Characterisation of Reproduction-Associated Genes and Peptides in the Pest Land Snail, Theba pisana
Source: PLoS One. 2016 Oct 5;11(10):e0162355. doi: 10.1371/journal.pone.0162355 (PMC5051934; doi:10.1371/journal.pone.0162355)

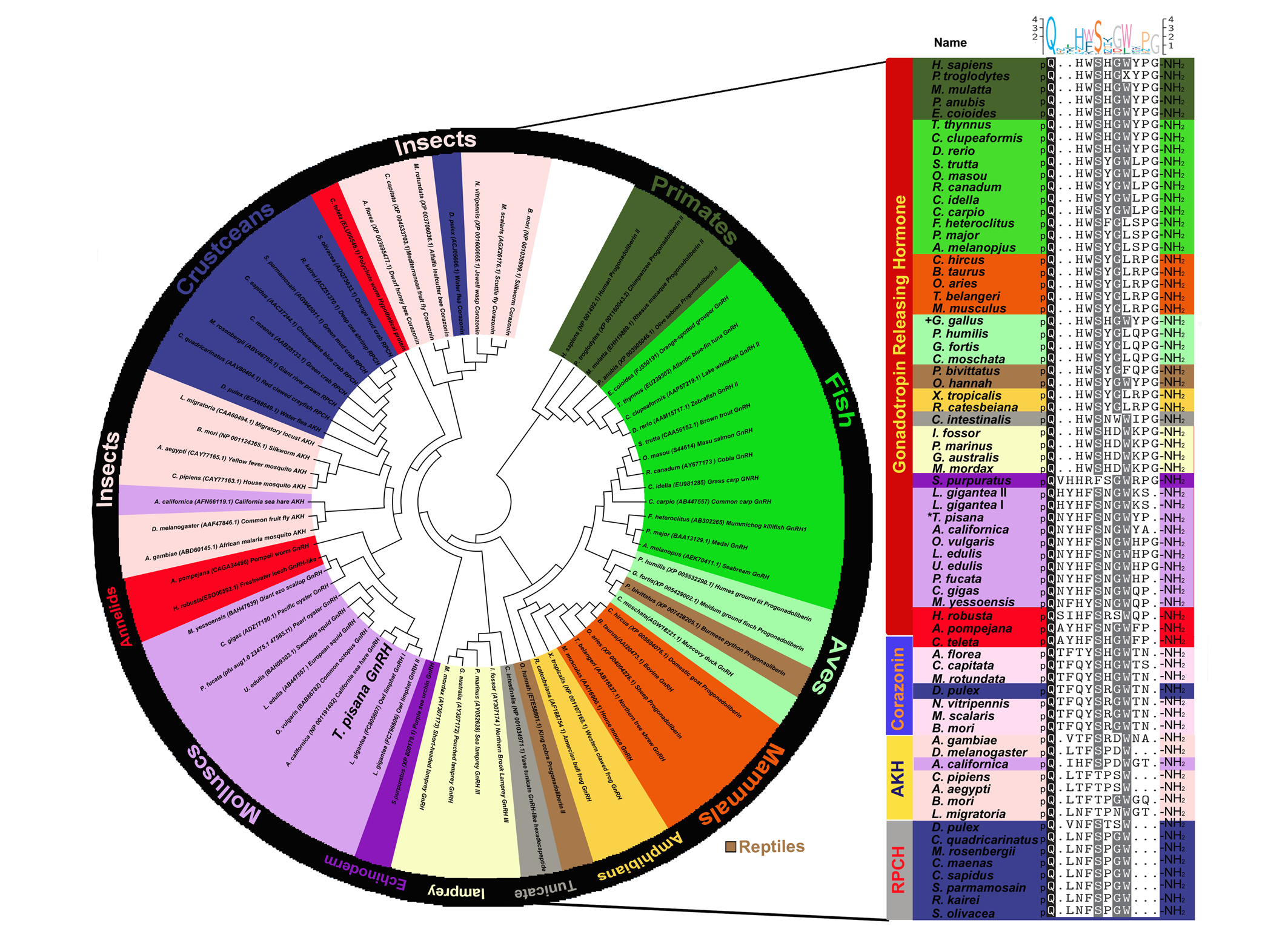

Supplement: S1 Fig — (TIF) [file pone.0162355.s001.tif]

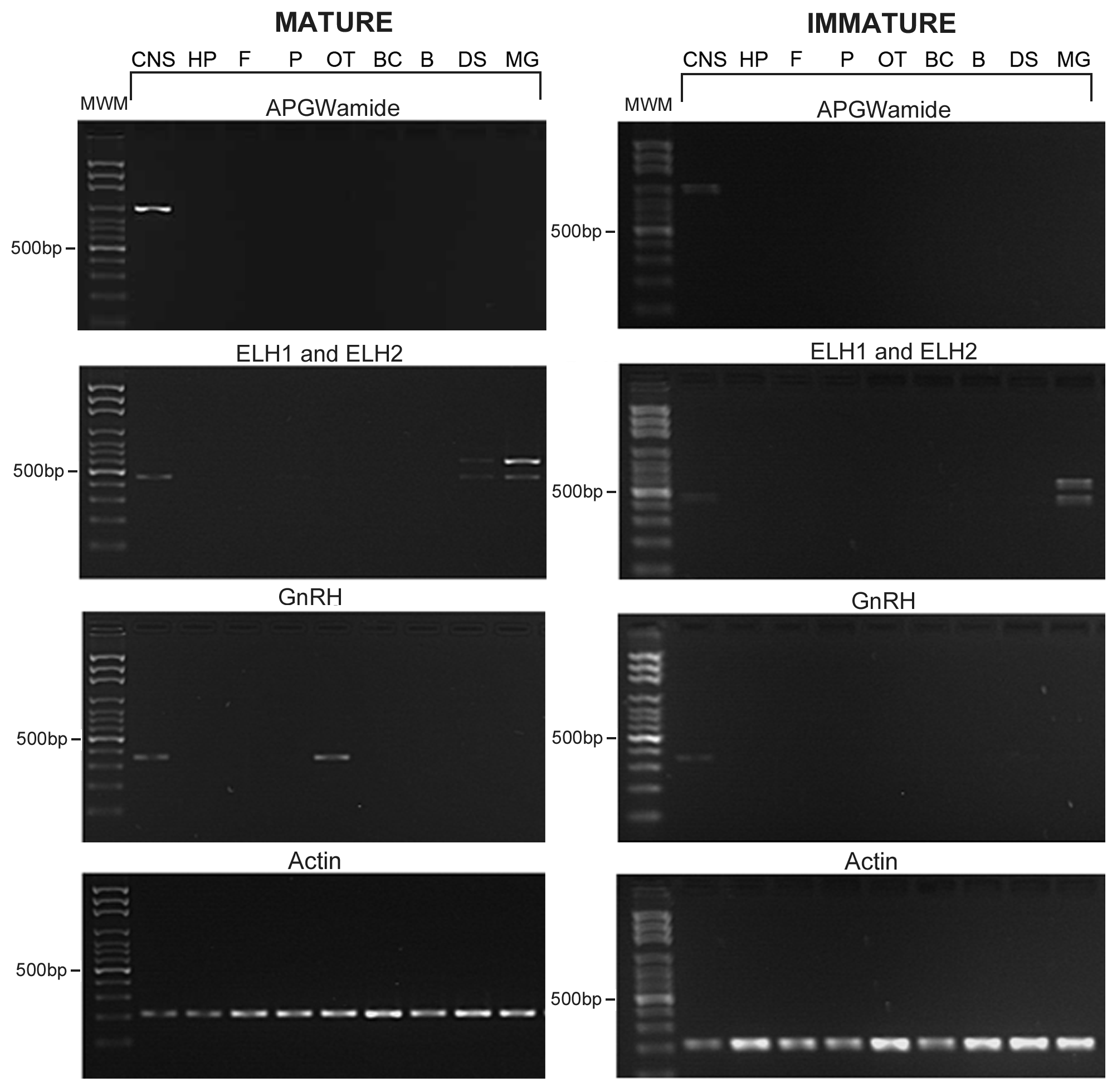

Supplement: S2 Fig — Representation shown in Fig 2. Tissues used include the: bursa tract (B), CNS (whole central nervous system ganglia), dart sac (DS), foot muscle (F), hepatopancreas (HP), mucous glands (MG), ovotestis (OT), penis (P). Tpi-actin was used as a positive control. (TIF) [file pone.0162355.s002.tif]

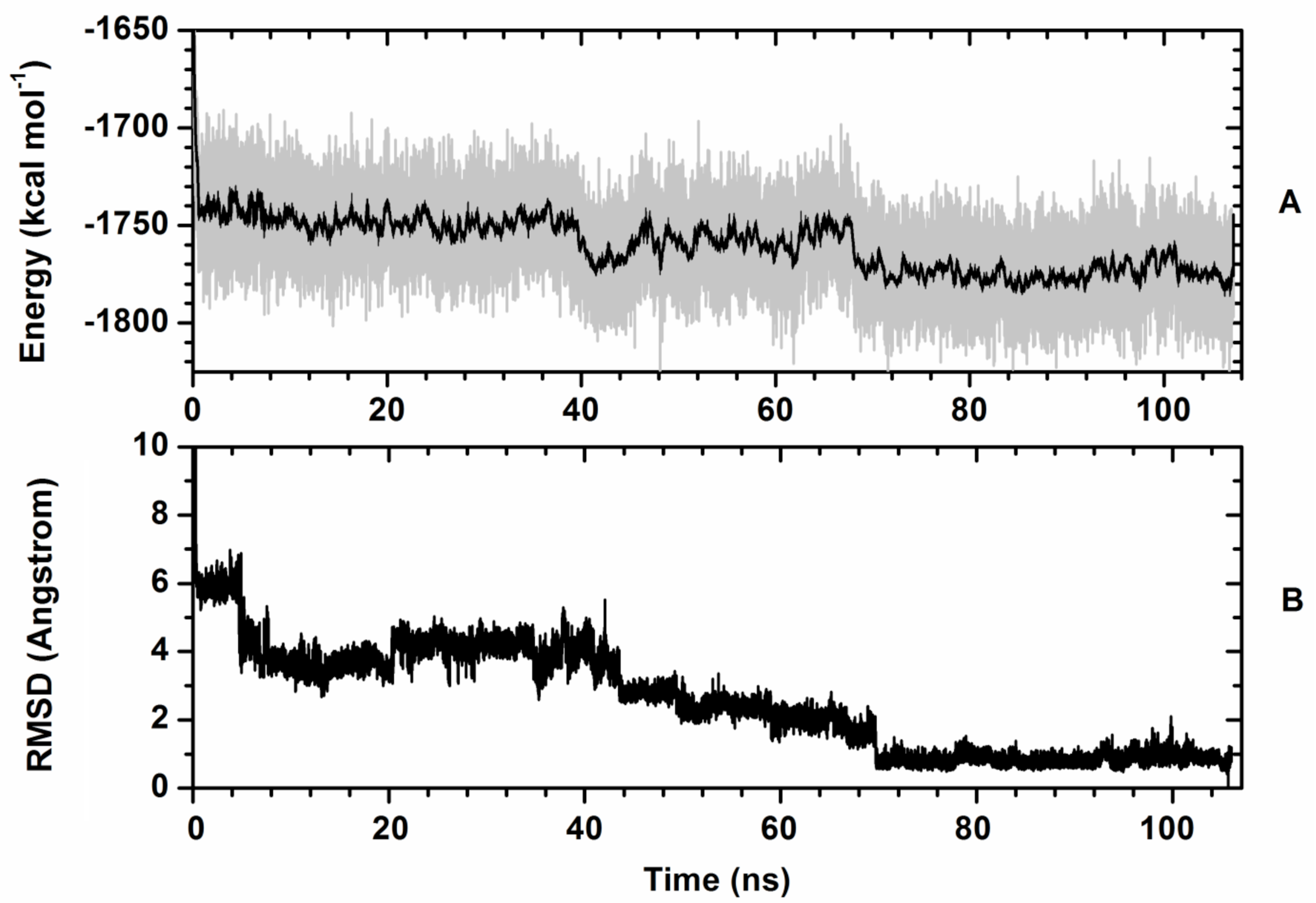

Supplement: S3 Fig — (A) Potential energy of ELH1 as a function of time during MD. The solid line is a running average over 50 ps. (B) Backbone RMSD during the same MD, compared to the lowest-energy conformation (the representative structure). ELH1 sequence: [p-] EAERDRRTWSISNALTVLTDMVVEHEQRRLAAEREALKQRLLELamide. (TIF) [file pone.0162355.s003.tif]
