# Supplementary material for: Characterisation of Reproduction-Associated Genes and Peptides in the Pest Land Snail, Theba pisana
Source: PLoS One. 2016 Oct 5;11(10):e0162355. doi: 10.1371/journal.pone.0162355 (PMC5051934; doi:10.1371/journal.pone.0162355)
Supplement: S1 Table — (DOCX) [file pone.0162355.s004.docx]

**S1 Table.** Primers used to amplify *T. pisana* genes.

| **Gene** | **Sequence** | **Expected size (bp)** |
| --- | --- | --- |
| Actin | 5’-CTCTCTGCTGTGGTGGTGAA-3’  5’-ATCCAGGCTGTCCTTTCCTT- 3’ | 212 |
| APGWamide | 5’-ATGTGTGCTAAACGCTCC-3’  5’-CCTTTGATCATCGGCACT-3’ | 966 |
| ELH | 5’-GATATCTTAAACAGCAATGTC-3’  5’-TAAACATTTTCTGAAGAGCCC-3’ | 452 |
| GnRH | 5’-ATGGTTTGTTGCAAGATG-3’  5’-CCATTTCCCCTCGGATTC-3’ | 354 |
